# Access to E‐Cigarettes Is Easy in a Regional Area in Australia: A Qualitative Study to Explore Young People's Views on Vaping

**DOI:** 10.1111/hex.70531

**Published:** 2025-12-19

**Authors:** Rishita Chandra, Seana Gall, Jasmine Angus, Suzanne Waddingham

**Affiliations:** ^1^ Tasmanian School of Medicine, University of Tasmania Hobart Tasmania Australia; ^2^ Menzies Institute for Medical Research, University of Tasmania Hobart Tasmania Australia; ^3^ Department of Health Tasmanian Government Hobart Tasmania Australia

**Keywords:** access to vaping, E‐cigarette use, regional setting, vaping, young adults, young people

## Abstract

**Objective:**

Working directly with young people to understand vaping use is necessary for developing meaningful strategies to support them to quit. We explored a range of aspects about the use of e‐cigarettes among young people in regional Tasmania, Australia, to inform local approaches to mitigate vaping.

**Setting:**

Tasmania is an island state with about two‐thirds of its population living in inner‐regional areas, and one‐third in outer‐regional, remote, or very remote areas. There are three regions: North, Northwest, and South.

**Participants:**

Eighteen‐ to twenty‐four‐year‐old Tasmanians who had tried vaping.

**Design:**

An exploratory qualitative study. Recruitment per region employed purposive snowball sampling. Data collection (2021–2022) included demographics, tobacco and e‐cigarette usage, participants' knowledge, beliefs, and access to e‐cigarettes. One‐on‐one interviews and group discussions were conducted using semi‐structured questions. Analysis used a combination of summative and conventional content analysis approaches to develop themes.

**Results:**

Twenty‐three qualitative interviews and group discussions were analysed, including 29 participants in the analysis sample, 13 females and 16 males (62% South, 24% North, and 14% Northwest). Six themes were developed: ‘switch, not quit’: smokers are not switching to e‐cigarettes to quit nicotine; ‘curiosity and social influence are key reasons why young people tried vaping’; ‘vaping is convenient‐ easy to get, easy to use and easy to hide’, ‘short‐term effects of vaping are experienced’, ‘knowledge about vaping is varied’; and ‘suggestion for actions from young people to address the issue of vaping’. The acceptability of vaping was high, and it was seen as safe.

**Conclusions:**

Our findings support the recently implemented vaping reforms for Australia and suggest that such reforms will be relevant in regional and rural areas. However, it is crucial to continue working with young people to ensure strategies are locally developed and meaningful.

**Patient or Public Contribution:**

Young people of Tasmania with lived experience were actively engaged throughout this research, to help us understand the underlying drivers of vaping in their communities. They have advised that if adults want to change the behaviour of young people, then we need to co‐design solutions with young people. More co‐design research is needed to incorporate the voices of young people and their ideas about prevention and cessation need to be considered alongside the implementation of regulatory changes.

## 1 Introduction

Vaping devices or electronic cigarettes (e‐cigarettes), a nicotine delivery system, were commercialised in 2003 [1]. The increasing prevalence of vaping has become an epidemic in some countries and a cause of global health concern [2, 3]. In particular, vapes are being used by many young people who have never smoked or smokers that have no intention of quitting nicotine. Of concern is recent evidence that vaping among non‐smoking young adults is associated with later smoking initiation [4].

While use is increasing across all age groups, in Australia, those aged 18–24 years have the highest lifetime use of vapes (26%) compared to other adult age groups in 2019 [5]. In 2022–23, current use ranged from 4% in Tasmania to 8% in New South Wales. In 2022–23, daily use increased in major cities (1.1% to 3.9%), inner regional (0.9% to 2.2%), and outer regional areas (0.8% to 2.5%) since 2019, while remaining unchanged and high in remote and very remote areas (3.7%) [6, 7]. The seemingly lower use of vapes in regional areas compared to major cities contrasts with the use of tobacco cigarettes, which is more common in regional or rural areas than major cities in Australia. Among young Australians aged 18–24 years, the majority of ever‐vapers in 2022–23 (57.6%) had never smoked before vaping, reflecting the trend toward uptake by non‐smokers [7]. Understanding the knowledge, attitudes, and beliefs of people who use e‐cigarettes in this age group will assist with the development of interventions to reduce uptake and increase cessation.

The evidence about young people's understanding about vaping in Australia is limited, particularly in non‐metropolitan areas. Recent studies highlight varied levels of access and knowledge about e‐cigarettes across different geographic settings. A recent study from Australia reported the ease of accessing vapes through informal channels [8]. A report by Cancer Council, New South Wales (NSW) reported that young Australians are easily accessing vapes from illegal sources [9]. A study focusing on metropolitan areas of Perth, Western Australia, indicates that youth in these settings have easy access to vaping products. Ease of access and the appealing nature of vapes were associated with a reduced desire to quit [10]. Similarly, a study from NSW, including participants from both metropolitan and regional areas, highlighted that young people were accessing vapes through various formal, such as petrol station, convenience store, or tobacconists; and informal channels, such as social media, friends, siblings, parents, or legal guardians. Many participants were not aware of the nicotine content in the vapes they had used [8]. A qualitative study with approximately 25% of participants from non‐metropolitan areas of NSW, reported prevalent positive vaping norms among adolescents, derived from social exposure and vaping promotion [11]. However, differences as per remoteness or rurality were not explicitly reported in these studies. Few of these existing studies have explored attitudes, beliefs of access within a regional or rural setting, despite the differing retail environments, social and economic drivers of health behaviours in such settings. Regional and rural areas differ substantially from those in metropolitan areas. Populations outside major cities often experience poorer health outcomes, shaped by persistent disparities in access to health services, including fewer primary care providers, longer travel distances, and limited specialist availability. These service gaps along with socioeconomic disadvantage and reduced availability of recreational opportunities, contributing to higher risk behaviours and health inequities. Distinct retail environments, with fewer formal outlets, licenced pharmacies, and greater reliance on informal supply networks, further shape patterns of vaping access and use [8, 12, 13]. Recent legislative changes in Australia have further tightened the regulation of vaping products. Nicotine‐containing vapes have always required a prescription, but until recently, non‐nicotine vapes (including single use, disposable devices) could be legally purchased through general retail outlets for people aged over 18 years. However, several random tests showed that vapes labelled as no nicotine did contain nicotine [14]. The 2023–24 national reforms addressed this anomaly by banning the retail sale of all disposable vapes, restricting flavours in pharmaceutical‐grade products, and introducing standardised packaging [15]. In Tasmania, the regulations are now stricter, from 1st October 2024, they introduced prescription‐only model for over 18‐year‐olds, while persons under 18 are prohibited from accessing vapes altogether, with no prescription pathway available [16, 17]. A recent qualitative study from Australia emphasised that alongside regulatory changes, it is crucial to support young people addicted to vaping or using them as coping mechanisms [18].

In the context of this rapidly emerging public health threat, we worked in partnership with the Tasmanian Government's Department of Health to do a qualitative study about e‐cigarettes in people aged 18–24 years. The research aimed to understand the use, access, and knowledge of e‐cigarettes to inform the development of interventions to address e‐cigarette use as part of the state's Tobacco Action Plan [19].

## 2 Methods

### 2.1 Research Design

This was a qualitative study using one‐on‐one and group semi‐structured interviews. This study was approved by the Tasmanian Human Research Ethics Committee (H0026260).

### 2.2 Recruitment and Participants

Tasmanians aged 18–24 years who could speak English and had tried or currently used e‐cigarettes were invited to participate. Purposive snowball sampling through existing youth networks was used to ensure representation across each region of Tasmania (north, south, north‐west). Recruitment aimed to achieve a diversity of opinions across relevant groups (e.g., socioeconomic status, gender, and region). Individuals not residing in Tasmania were excluded. Informed consent was obtained from all participants. As the data collected were qualitative, no minimum sample size was required for it to be methodically robust. However, data collection continued until data saturation was reached [20].

### 2.3 Description of Terminology Regarding E‐Cigarettes or Vaping

Throughout this manuscript, the terms *e‐cigarette* and *vape* are used to refer to the same battery‐powered devices that heat a liquid to produce an aerosol for inhalation with or without nicotine, which users inhale. E‐cigarettes are the language that was, and still is, used to describe these electronic cigarettes. However, as the use among young people increased quickly after 2019, especially the use of single‐use devices, vapes and vaping were used more commonly among young people. Therefore, this became the language more commonly used in our research and the literature. Hence, we have interchangeably used these terms based on context.

### 2.4 Data Collection

Data collection commenced in 2021 and was completed in 2022.

#### 2.4.1 Semi‐Structured Interviews

Interview questions included tobacco and e‐cigarette use, access, knowledge, and beliefs about vaping. There were two ice‐breaker questions and ten base questions (see Supporting Information 2). Same semi‐structured questions were used for both the individual and group interviews. As an icebreaker, and to better understand how young people refer to e‐cigarettes, we asked each participant what language they used to describe e‐cigarettes and smoking. Interviews were conducted individually or in groups, either online or face‐to‐face, depending on the preferences of participants. The anticipated time required for individual interviews was about 30 min and for group interviews about 60 min. We provided participants with information about the research and the team prior to obtaining consent. Data collection continued until data saturation was reached. On completion of the interview, each participant was provided with a gift card worth A$30 as a reimbursement for their time.

### 2.5 Data Analysis

Descriptive statistics were used to report demographic information. A combination of deductive and inductive qualitative analysis was used to analyse the data. Conventional content analysis was used to analyse responses to each question and overall responses. Conventional content qualitative analysis is an inductive approach to develop themes from data using direct coding [21]. Summative and Conventional Content Analysis methods [21] were utilised to identify common themes and word patterns using N‐Vivo version 20.6. We conducted individual and group interviews between June and August 2021. The audiotaped interviews were transcribed and interpreted by one researcher (SW). The analysis was a mix of closed and open coding. between June and August 2021.

## 3 Results

A total of 23 interviews with a combination of 17 individual and six group interviews were conducted, involving 29 young Tasmanians. However, one participant's record was removed during data cleaning as their age was outside the eligibility criteria. After excluding one participant due to their age, the demographic profile of the remaining 29 participants comprised 13 females and 16 males with a mean age of 22 (SD 1.86) years. There were two participants per group, except Group 2, which comprised three participants. Distribution by region included seven participants from North, 18 from South and four from Northwest Tasmania. From the icebreaker questions, it was clear that young people refer to e‐cigarettes as vapes, and rather than smoking, they say vaping or having a puff. Most participants reported starting to vape one to 4 years ago aged around 18–19 years old.

The thematic analysis of the interviews yielded six major themes, some with sub‐themes. The themes include ‘switch, not quit’; ‘curiosity ‐ stuff it, why not’; ‘convenience ‐ easy to get, easy to hide’, ‘short‐term effects are experienced’, ‘knowledge is limited’; and ‘suggestion for actions’. The following sections describe these themes in detail with representative quotes for illustrative purposes.

**Theme 1 – ‘Switch not quit’: Smokers are not switching to e‐cigarettes to quit nicotine**

Vaping was identified by many participants as a healthier alternative to traditional smoking by many participants. Among those who smoked, vaping was often adopted as a strategy to quit or reduce tobacco use. However, many said they continued vaping as it was a healthier alternative and cheaper than smoking. Other reasons to initiate vaping included the tolerable smell than tobacco smoke, and it was much easier to be discrete when vaping in non‐smoking areas. They stated:

It was not only much nicer than a cigarette but um, I was under the impression that it was also much healthier.

(Individual Interview 13)

That's how it got brought in, it's like a healthier option to smoking, and then it took off on its own thing. And more people started vaping that maybe would have never started smoking.

(Group Interview 1, *n* = 2)

Whereas, with cigarettes, when you light it up, within half an hour you can smell cigarettes and, like, giving people who vape, you cannot smell it on them.

(Group interview 2, *n* = 3)

**Theme 2‐ Curiosity and social influence are key reasons why young people tried vaping**

In our study, respondents frequently mentioned ‘curiosity’ as a primary reason for trying vaping. Many individuals who had never smoked cigarettes and had no intention of doing so, started vaping out of curiosity and finding it to be a cool and fun activity. Young people were drawn to vaping as their friends and peers were doing it, and vaping was perceived as a trendy and socially acceptable behaviour.

I was like oh well, now is the time to discover what it's like because I've never smoked or anything before. But I just was curious.

(Group Interview 1, *n* = 2)

And most of them, you know, they are vaping because they think that it's really cool, or something like that. So, they don't smoke in their past. They just vape because many people are vaping.

(Individual Interview 8)

So, it's sort of about fitting in because everyone else is doing it.

(Individual Interview 3)

**Theme 3‐ Vaping is convenient ‐ Easy to get, easy to use and easy to hide**

Our analysis identified convenience as a common theme with three sub‐themes: accessibility, acceptability, and versatility, irrespective of the respondent's smoking history.

### 3.1 Vapes Are Easily Accessible

Access to nicotine and nicotine‐free vapes was reported to be easy in Tasmania. Most purchased them online, through local suppliers (friends or dealers) who buy disposables in bulk. In addition, there have been those who purchased nicotine to ‘spike’ the vapes, made their own liquid, bought an online prescription, and accessed through various places/social media using a code name.

And there's this one guy … and he does, like, drop‐offs and pick‐ups, and he always has, like, a bajillion of them.

(Individual Interview 5)

There are also a few clubs I think that sell them so when people are out at town, they can go in…. You have like code words for them.

(Individual Interview 17)

So, the company that I'm using for the vapes, they have a contract with a pharmacy. Yeah, so everything is done on their website.

(Individual Interview 8)

It's on Snapchat. But a lot of his is, like, through word of mouth

(Individual Interview 5)

### 3.2 Vapes Are Socially *Acceptable*

In our study, participants talked about vaping being more acceptable than smoking a cigarette, as well as the ease of being discrete while vaping in smoke‐free areas.

It's cheaper, it's accessible, it's more socially acceptable.

(Group Interview 2, *n* = 3)

Yeah, it's like no smoking areas, you'll see people vaping all the time because it's not considered the same thing.

(Group Interview 2, *n* = 3)

It's also an interesting thing with, like, law enforcement, like police officers and stuff like that, they still look towards cigarette smokers a lot more than vape ‐ people who vape.

(Group Interview 2, *n* = 3)

### 3.3 Vapes Are Versatile and Convenient to Use as Compared to Traditional Cigarettes

Participants found e‐cigarettes convenient to use because of their perceived versatility. Young people identified it as easy to use and easy to hide. Several participants also identified e‐cigarette use as appealing in terms of smell, flavour and low cost.

But it's easy to do because all you do is just pick it up. Press a button for a second. Yeah, you've got your hit. Yeah. Yeah, it's very quick.

(Individual Interview 14)

It's also younger kids who are getting into vaping, it's so much easier to hide than, say, a pack of cigarettes…

(Group Interview 2, *n* = 3)

Like, some are as small as a pen. Like, the one I've got is pen‐sized.

(Group Interview 2, *n* = 3)

It doesn't stick to them, wherewith someone who smokes, it's in their clothes and their hair. When I used to smoke, I'd be like, my hair stinks. This is gross. Whereas I'm vaping everywhere, and I don't smell anything.

(Group Interview 2, *n* = 3)

…it's really interesting because one of the things that made me feel like I could vape a lot more than smoke is just cost.

(Group Interview 2, *n* = 3)

**Theme 4‐ Short‐term effects of vaping are experienced**

It is noteworthy that young people who had been former smokers or had never smoked, encountered many pleasant and unpleasant effects while using e‐cigarettes. Some reported feeling calm and relaxed after vaping. The participants also liked the head spins; they experienced immediately after inhaling.

It just gives a cool and relaxing feeling. So, I tend to use it more when I'm stressed

(Group Interview 3, *n* = 2)

Just, I guess, in general, stressful, more stressful environments that you're placed in and whatnot? Oh, you'll find yourself toking on it a bit more than usual, … It kind of knocks you back down,… The good feel. I like it because it's more of a calming agent.

(Individual Interview 14)

I was so delighted by the headrush that came up with the use of the substance.

(Group Interview 5, *n* = 2)

I remember getting, like, the head rush and getting really, like, lightheaded. And it almost feels like, like a weight has been lifted off you. It was so weird.

(Individual Interview 5)

Besides the above‐mentioned pleasant feelings, participants also reported having adverse effects: ‘Let's say I've been vaping for more than 15 times a day for a long period within each session, I realise that by the evening I will have some migraines’.

(Group Interview 5, *n* = 2)

Layer of phlegm kind of through your throat that you cough up all the time

(Group Interview 2, *n* = 3)

But vaping ‐ yeah, vaping just tended to make me feel, make my lungs feel heavy, is what I found most.

(Group Interview 2, *n* = 3)

**Theme 5‐ Knowledge about vaping is varied**

Participants had limited knowledge about the contents of e‐cigarettes, the health effects, and the laws, with most focusing on nicotine and available flavours. Participants also had varying perceptions about the health effects of vaping with some believing that they were a safer alternative to smoking. Additionally, it was observed that while most participants were aware that there are laws, their understanding was restricted. Many noted that people vape in smoke‐free areas and are easy to disguise.

Participant's *statements on what is in a vape:*

Definitely wondering, like, what sort of what, how did they get the flavour in there and things like that.

(Individual Interview 9)

I only ever really looked at whether they contain nicotine or not and just the flavours.

(Group Interview 4, *n* = 2)

And I don't think personally that, like, vaping is as bad as cigarette smoking. So, I think we should allow it to sort of be a little bit… Yeah, not prohibited, I suppose!

(Individual Interview 9)

I tried to do some, you know, research a bit online. You know, some articles say that they are bad. Some say that you know, they don't know the long‐term effects of vaping. And some articles are really positive about vaping.

(Individual Interview 8)

Until they scientifically prove that it'll kill me, I'm going to keep vaping.

(Group Interview 6, *n* = 2)

Participant's statements on vaping laws:

I don't know about any vaping regulation laws and where you can and where you can't.

(Individual Interview 14)

In regard to smoking and vaping stuff, never really have read up on the laws because I was – I've only become an adult recently, legally at least.

(Group Interview 1, *n* = 2)

But it was just something we never did the research, is this legal, is it illegal? It didn't matter. You just did it …

(Group Interview 1, *n* = 2)

**Theme 6 – Suggestions for action from young people to address the issue of vaping**

There was a range of ideas from young people about action to address e‐cigarette use (see Supporting Information 1). Young people thought messages to prevent uptake or increase cessation should be relevant, non‐judgmental, informing and designed for young people. For example:

It's about, instead of enforcing these are really bad for you, it's about trying to understand why that person is doing what they're doing, and despite it being bad, why they feel like they need it more.

(Group Interview 1, *n* = 2)

Young people also suggested that interventions should be co‐designed with them; otherwise, there is potential risk for the campaigns to not work. They stated:

That's like it could be pretty important to be youth‐designed or youth‐referenced, like us, or we can test it because sometimes what people think will work can just totally miss the mark.

(Group Interview 1, *n* = 2)

Young people suggested a number of other avenues to address e‐cigarettes. Some people discussed harnessing the potential environmental effects of e‐cigarettes in terms of single‐use plastics in disposable devices and inappropriate disposal of battery‐operated e‐cigarettes. There were thoughts about putting labels on e‐cigarettes and/or packaging listing ingredients to increase the knowledge of users. There was also interest in greater promotion of the mechanisms for the prescription pathway for current smokers of regular cigarettes who want to use e‐cigarettes to quit. The quote verbatim from young people is mentioned in Supporting Information 1.

## 4 Discussion

The widespread prevalence of vaping poses a significant public health concern that transcends regional and geographical boundaries. Given the harms and prevalence of vaping among young people, limiting their access, and providing awareness about the harmful effects will be pivotal for e‐cigarette control. At the same time, care needs to be given to youth‐focused cessation support as evidence reports that many young people are feeling addicted to vapes [22, 23].

This study offers significant insights into the use of vapes among young people in the regional setting of Tasmania. It reveals multiple drivers of use, varying perceptions of risks and health‐related harms, and suggesting that accessing e‐cigarettes is easy for young people in Tasmania. Most people, particularly people who did not smoke combustible cigarettes, used disposable, flavoured devices. There is considerable uncertainty among young people regarding the harms of e‐cigarette use, but people broadly support taking action to restrict access and acknowledge the potential threat to health. These findings support local and national action on e‐cigarette use by young people, considering the recently implemented policy reforms. As discussed in detail below, the findings are mostly consistent with existing studies conducted in both regional/rural and urban settings in Australia, with some differences, highlighting the potential ability to use interventions to reduce vaping uptake and increase vaping cessation across regions.

The ease of accessibility of vaping devices emerged as a crucial factor in Tasmania. In consistency with the literature largely based in metropolitan areas [8, 24], many young people purchased their e‐cigarettes from local dealers, online and through friendship groups. The informal networks, such as peer groups and local suppliers, were highlighted as predominant sources of e‐cigarette products. In our study, young people revealed that they can easily access e‐cigarettes (with and without nicotine) using code names online but also in some retail and hospitality environments. A study by Watts et al. which comprised 80% participants from metropolitan areas of NSW, reported that young people accessed e‐cigarettes both through social and commercial channel with majority accessing through informal channels [8]. This is consistent with another study that found that young people commonly get e‐cigarettes through purchases from stores or online, as well as by buying directly from peers [25].

However, differences emerge in the relative formal retail access. Watts et al. [8] reported that while most young people obtained vapes through informal networks such as friends or siblings, nearly one‐third (31%) purchased their most recent device from retailers, including petrol stations, tobacconists, and convenience stores, with smaller proportions using social media (9%) or websites (7%). In contrast, our Tasmanian participants described formal retail purchasing as far less common, relying predominantly on local informal networks. This indicates that although informal supply is a key source in all settings, it may be even more common in regional areas where young people have fewer opportunities to buy vapes from retail outlets.

Although obtaining a prescription from a general practitioner for nicotine‐containing vape products was mandated by the legislation in 2021 [26], the current study reflects that young people managed to purchase e‐cigarettes containing nicotine without having a prescription. Those who had a prescription mostly obtained it online. This discrepancy highlights a significant loophole in the enforcement of the vaping‐related regulations. In response to these challenges, the Australian government has implemented the Therapeutic Goods and Other Legislation Amendment (Vaping Reforms) Bill 2024. The reform includes recent changes designed to strengthen the e‐cigarette‐related laws and to cease the loopholes that allowed unauthorised access to nicotine‐containing vapes [27]. However, the significant ‘black market’ in e‐cigarettes will need to be addressed to ensure the effectiveness of the recently announced federal restriction of e‐cigarettes to those with prescriptions.

The current study reflects that young people started vaping for varied reasons including, but not limited to, tobacco smoking cessation. Interestingly, many tobacco smokers were not intending to then give up smoking nicotine, it was a switch to e‐cigarettes rather than a progression to stop the habit all together. Several young people, including those who have never smoked, started vaping out of curiosity, which aligns with the findings of some qualitative studies conducted in the United Kingdom [28] and Australia, representing participants from metropolitan areas [10]. Consistent with our findings, the availability of numerous flavours has been found to drive youth e‐cigarette use [29]. The factors like peer influences, social acceptability and vaping being ‘fun & cool’ have also somehow contributed to its increased popularity among the youth, consistent with the findings of other studies [30, 31]. Young people were not concerned with the nicotine content of an e‐cigarette or how much they were consuming, similar to the findings of a qualitative study of a friendship group [28]. This suggests that there is likely to be a large group of young people addicted to nicotine through vapes. There is a lack of evidence‐based recommendations for vaping cessation among young people [32]. The likely reduction in the availability of e‐cigarettes in Australia will mean an increase in the need for youth‐centric cessation services.

An Australian study conducted on young people who were never smokers, reported a significant association between e‐cigarette use and susceptibility to start smoking cigarettes in the future [33]. Another study from New South Wales, Australia, conducted on young people, concluded that vaping and smoking are significant risk factors for one another, suggesting a non‐linear relationship between e‐cigarettes and traditional cigarettes [8]. In accordance with their findings, we also found that young people started vaping for various reasons, and smokers and non‐smokers who started vaping indicated dual use or switching of products.

The present study revealed varied knowledge and belief attributes related to e‐cigarettes among young adults. As reported by some qualitative studies from the metropolitan areas in the USA [34, 35], the current research also found that young people were not fully aware of the severity and negative consequences of e‐cigarettes. Similar to the findings of other studies [36, 37], participants of our study also perceived e‐cigarettes to be ‘safer’ and ‘healthier’. Although the knowledge related to its adverse effects and regulating laws was limited, participants reflected some understanding towards vaping being a health concern. Another study from Australia by Jongenelis et al., reported limited awareness about vaping‐related harms [38].

Similar to our findings, the literature [39, 40] has reflected that e‐cigarettes are being used by young adults to cope with stressful circumstances. The *head rush* and *feeling calm or relaxed* appeared to be positive reinforcement for vaping in our study. The ease of access [41] and hiding e‐cigarettes are added advantage which allows young people to vape anywhere, sometimes in social gatherings and sometimes in boredom. Consistent with the findings of another study [42], participants reported some short‐term effects like breathlessness, coughing, a film on the throat, migraines, and dry mouth. Contrary to their findings [42], we found that the reported short‐term effects did not discourage e‐cigarette use among young people, rather the lack of evidence about its long‐term harms was quoted as one of the reasons to continue vaping. The qualitative theme identified that many knew there were chemicals in the devices but did not express concern about the potential long‐term health effects. The reports of e‐cigarette use providing a calming effect may in fact reflect that many young people are addicted to nicotine, with the use of the e‐cigarette reducing withdrawal symptoms, which may be experienced as anxiety or ‘stress’.

The recent legislative changes implemented by the Australian government to prohibit recreational vaping [43], is a profound step towards e‐cigarette control. Our findings support many of these reforms, particularly around removing disposable and flavoured devices. The findings from a report published by the Cancer Council Australia also supported the recent vaping policy reforms, suggesting that it can reduce the accessibility of vaping products [44]. Of note is the large black market for e‐cigarettes, outside of licences retailers. Our findings support the reforms to strengthen enforcement around the importation of devices. Participants in this qualitative study clearly articulated that they should be involved in the response to vaping. Considering young people's views in the policy reforms to address vaping will play a pivotal role in its successful implementation, including buy‐in from this target group to bring about behaviour change among young adults. As evident from the findings of our study, there is a need to co‐develop the messages and policies meant to spread e‐cigarette awareness among youth. The current study highlights the importance of exploratory research with young people. Focusing on the views of young people and what they need and seek in an intervention targeted at youth e‐cigarette control is of utmost importance. Hence, we advocate the need to co‐design behavioural and educational interventions for preventing young people from taking up e‐cigarettes and in designing interventions to promote the cessation of e‐cigarettes.

This study was subject to a few limitations. As the sampling technique was purposive, it limits its generalisability. Due to time constraints, the qualitative data were coded by only one researcher. The data for this study were collected in 2022, federal and state legislation around e‐cigarettes is rapidly evolving and were different then from now. Our qualitative data collection and analysis did not allow exploration of some potentially relevant quantitative information, such as purchasing behaviour and access to vapes. The strengths include the entirely regional sample, including a diverse population across the state.

## 5 Conclusion

E‐cigarettes are widely available in Tasmania, a regional state of Australia, with use among varied groups of young people for a variety of reasons, mostly related to socialising, curiosity and a sense of ‘fun’. Using e‐cigarettes solely as a means to quit smoking was not common; instead, young people also reported initiating vaping for other reasons noted above. Although young people had some understanding of the short‐term harms, their knowledge was limited about the long‐term effects. There was a lot of uncertainty about e‐cigarettes in general. Our results were mostly similar to studies focused on people in metropolitan areas. This research creates a comprehensive baseline of information that can be used for monitoring changes in knowledge, beliefs, access, and use of e‐cigarettes over time. Our findings support the recently proposed, world‐leading reforms in e‐cigarettes for Australia, suggesting they will be relevant and effective in regional and rural areas.

## Author Contributions

**Rishita Chandra:** writing – original draft, writing – review and editing. **Seana Gall:** conceptualisation, supervision, writing – review and editing, funding acquisition. **Jasmine Angus:** writing – review and editing, funding acquisition. **Suzanne Waddingham:** conceptualisation, investigation, formal analysis, supervision, writing – review and editing, funding acquisition.

## Ethics Statement

This study was approved by the Tasmanian Human Research Ethics Committee (H0026260).

## Conflicts of Interest

The authors declare no conflicts of interest.

## Supporting information

Supporting Material.

## Data Availability

The data that support the findings of this study are available from the corresponding author upon reasonable request.
