# Supplementary material for: Access to E‐Cigarettes Is Easy in a Regional Area in Australia: A Qualitative Study to Explore Young People's Views on Vaping
Source: Health Expect. 2025 Dec 19;28(6):e70531. doi: 10.1111/hex.70531 (PMC12715590; doi:10.1111/hex.70531)
Supplement: Supplementary file 1 — Supporting Material. [file HEX-28-e70531-s001.docx]

**Supplementary Material 1**

Young people’s thoughts (verbatim) about what to consider when communicating to young people about vaping.

| - *That’s like it could be pretty important to be youth designed or youth referenced, like us, or we can test it because sometimes what people think will work can just totally miss the mark.* - *Some of those myths more, about how even the nicotine-free ones contain nicotine. That might kind of shock some people, that oh shit, I can’t just keep smoking those for the fun of it. Or the effects of still inhaling and all that crappy – yeah, so maybe feeding some of those myths.* - *And with the cost thing, could you do a thing where so many vapes equal, I don’t know, something that you’d like to buy?* - *I don’t think typically young people, I don’t think, need the energy from nicotine.* - *Something else could be about the amount of money you save by quitting* - *I think the most powerful deterrent is just to say that it’s the same as smoking, it’ll do the same damage. Because the information about smoking is out there and it’s well known, and I feel like almost trying to create a separate thing with vaping it just – it’s starting from the ground up again. You’re trying to build up the same stigma, but if you just tie it into that same one, say it’s the same thing, people will go that’s not good.* - *I think with the long-term stuff as well; I think they miss the mark with the cigarette packaging. I think that’s a big thing for young people particularly, because it’s [inaudible] 00:34:57 just trying to get through the next day or week or whatever. We can’t think about 10 years in the future. Our existence has already been so stressful, with one disaster after another. The world’s going to be flooded or on fire or something.* - *I think there should be more realistic information spread about vaping and you've got cigarettes here in Australia, which you've got really horrible pictures right on the front of it, but people really still don't understand what vaping does and how dangerous it really can be for some people, people who end up with lots and lots of fluid in their lungs from vaping end up going to hospital* - *Like, it's just a thing of, socially you hear about it and it's just this kind of positive thing, oh, it helps you quit smoking. It's got different flavours. You always hear the positives about it and you don’t ever hear the negatives like smoking cigarettes.* - *There's so many different factors that come in to try and help young people get off nicotine. It’s not just, stop smoking.* - *So, it's like a thing of - instead of telling me, everything I'm doing is wrong here and I'm going to die from this, why don't you just kind of sit back and actually look at why you're doing this in the first place and healing that part of you that says, I need this nicotine, I need this, instead of just being like, just get off nicotine. Just do this and do this. Because that's never going to solve anything.* - *I think it's important to know that probably with our generations these days the scare tactic doesn't really work.* - *We know that this isn't good for us, but it's the fact of all we're getting from the people is, this is going to kill you. This is horrible. It’s not like caring, "what's going on? Why do you need this? Why do you feel you need this?" It's just, "This is horrible for you. Why are you being such an idiot?"* - *Yeah, so I think people who - I think if we want to encourage people to get off vapes and nicotine for us in general it's very much about encouragement and support, and I don't think forcing people to accept that it's bad is ever going to work because I think everyone knows that it's bad. It's about convincing people that they will feel better without it.* - *It’s about, instead of enforcing these are really bad for you, it's about trying to understand why that person is doing what they're doing, and despite it being bad, why they feel like they need it more.* - *If someone for instance came up to me and started berating me about how bad cigarettes are for my health and how I need to stop, the only thing I'm thinking about is “You don't understand. You don't understand what it's like for me, so I'm not going to listen to you.”* - *Everyone smokes for a reason* - *I think that's just a general thing with young people is they want to feel like they can relate to something. If you've got people that are not relatable, I think you really struggle to get young people to listen.* - *So, they should design a very interactive website* - *Until it’s scientifically proven, I don’t really want people coming up and telling me that stuff. I am aware that it’s not good for you. Any inhaling some sort of something into your lungs besides air isn’t really good for you anyway. So, I already knew that.* - *I’m not really one for social media, but social media, definitely. Popping it on social media would be – yeah, a good way of doing it.* - *But also, it’s the lack of foundation, because someone saying it’s worse than smoking when there’s no proof of it is a bit also like – you can’t prove it. So, definitely, need good foundation, I think, on it, if you’re trying to get it out there a bit. Because I think that’s the one thing with vaping that I find is probably the biggest struggle, is that it - like I said, there’s not a lot on it yet. It’s not as known as cigarettes. So, there’s not as much research, there’s not as much proof.* - *There’s not like – no, 100 years of research on cigarettes, where – probably within the first sort of few years with vaping, and we’re only just starting to look deeper into it now. So, I think it is something that just kind of does need the time and a bit of effort put into it.* - *But reaching out for young adults or younger – older teens, I should say – young adults and older teens – is, like I said, definitely social media. It’s the only one I can actually think of, because the majority of the people around that age in that generation is just all on social media and everything that they’re influenced by is pretty much social media, sadly.* - *Non-judgemental. Don’t – because like I said, people that smoke and people that vape, they know it’s not good for them, so – when people are judgemental are about it, it kind of just makes you want to put your middle finger up at them, because – you’re aware that it’s not good for you, and there’s no need for the judgement. So, definitely come at it in a way that it’s not judgemental, it’s very positive.* - *The younger adults and the older teens, because it’s more – vaping’s more of a thing for people around that age.* |
| --- |

**Supplementary Material 2**

Interview Questions

1. Icebreaker Question 1 – Before we start, I’m interested to learn from you what people call an e-cigarette? Is there a particular name people use here in Tasmania?
2. Icebreaker Question 2– Can you share your story about the first time you tried an e-cigarette?
3. Can you talk about your use of e-cigarettes? How has this changed over time?
4. Where do you get e-cigarettes from?
5. What do you know about what is in an e-cigarette?
6. What do you know about the law around e-cigarettes in Tasmania?
7. Can you tell us about how you use e-cigarettes? (Prompts - parties, locations, chain etc.)
8. What are your thoughts about the use of E-cigs versus other cigarettes? (tailor made or rolled)
9. What are the reasons why you use e-cigarettes?
10. Have you experienced any bad effects from e-cigarettes?
